# Supplementary material for: Amphioxus adenosine-to-inosine tRNA-editing enzyme that can perform C-to-U and A-to-I deamination of DNA
Source: Commun Biol. 2023 Jul 18;6:744. doi: 10.1038/s42003-023-05134-0 (PMC10354150; doi:10.1038/s42003-023-05134-0)
Supplement: Supplementary file 2 — Supplementary Information [file 42003_2023_5134_MOESM2_ESM.pdf]

Supplementary Information

Amphioxus adenosine-to-inosine tRNA-editing enzyme that can perform C-to-U and A-to-I deamination of DNA

Gao et al.

Supplementary Figure 1

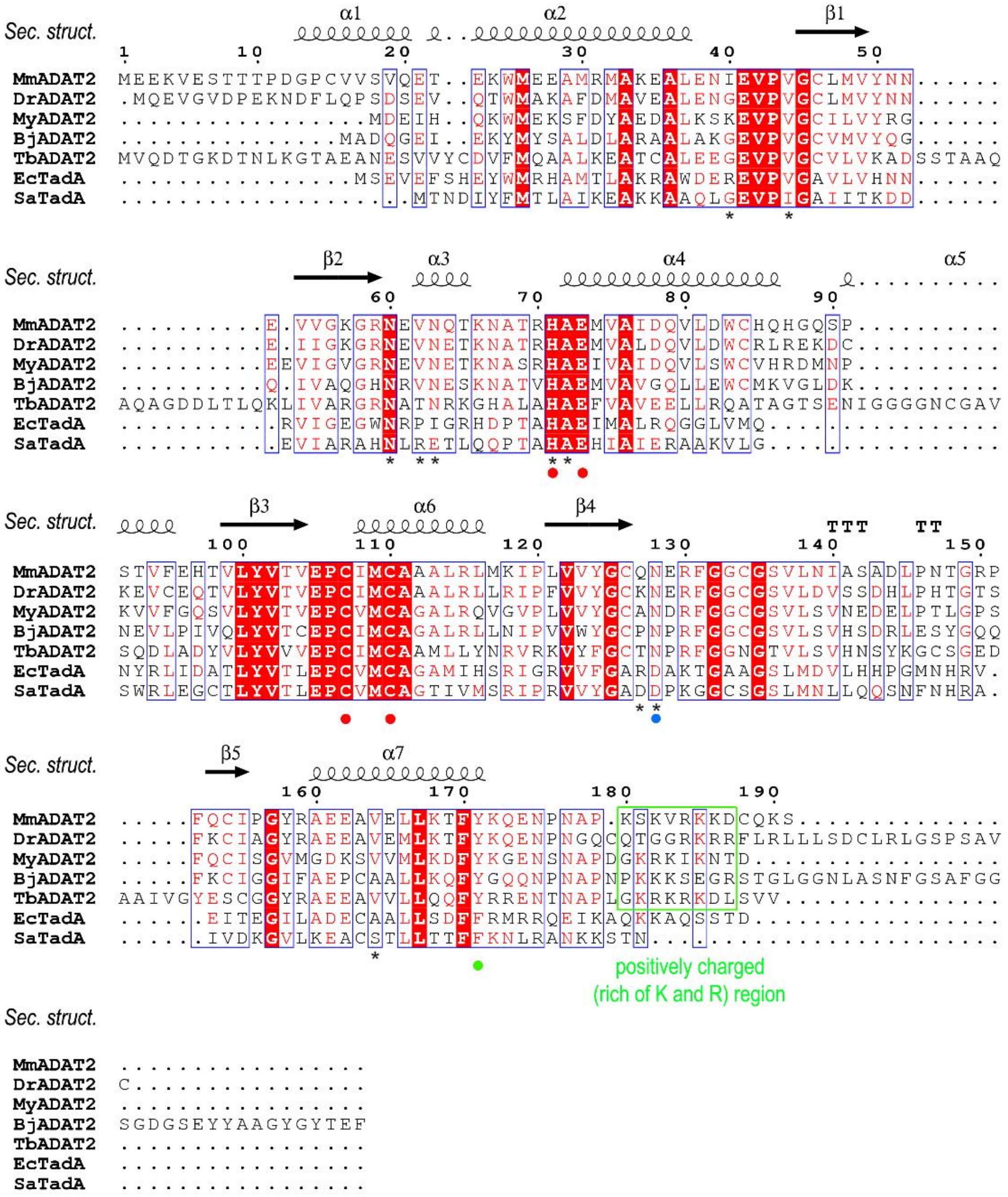

Multiple sequence alignment of ADAT2. The ADAT2 from *Mus musculus* (MmADAT2, NP_080024.3), *Danio rerio* (DrADAT2, XP_005160675.1), *Branchiostoma japonicum* (BjADAT2), *Mizuhopecten yessoensis* (MyADAT2, XP_021373128.1) and *Trypanosoma brucei* (TbADAT2, RHW71099.1), as well as their prokaryotic ortholog TadA from *Escherichia coli* (EcTadA, VWQ04824.1) and *Staphylococcus aureus* (SaTadA, WP_000180290.1) were included. The secondary structure elements were labeled above the sequences (based on MmADAT2). The asterisk indicated the key residues for recognizing nucleotide substrates. The red dots indicated the residues of active site. The blue dots indicated the residue D108 in EcTadA (equivalent to N113 in BjADAT2) that was shown to be a determinant for targeting RNA or DNA. The green dots indicated the residue F149 in EcTadA (equivalent to Y156 in BjADAT2) that was important for the deamination activity. The positively charged (rich of K and R) region was indicated by the green box.

Supplementary Figure 2

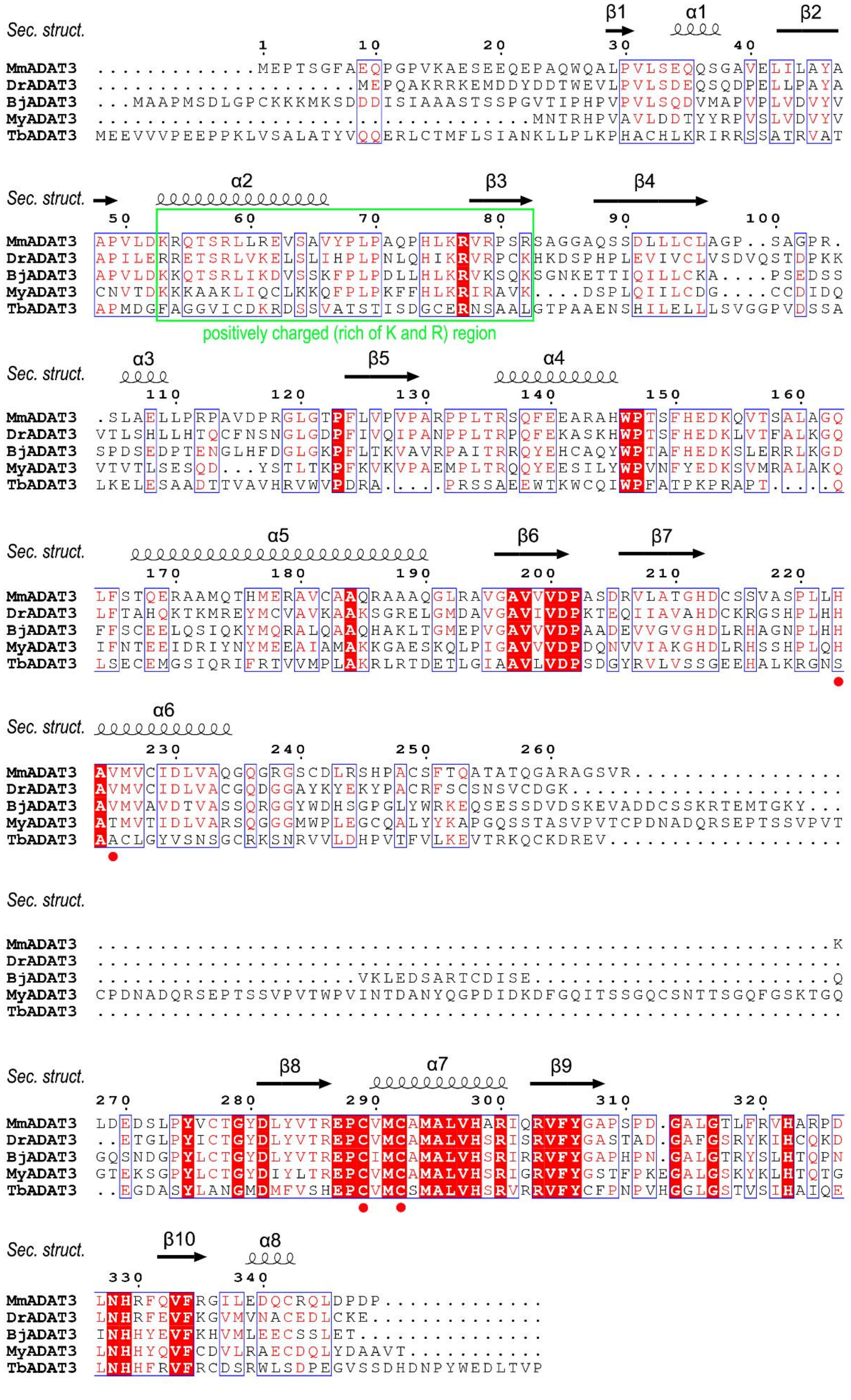

Multiple sequence alignment of ADAT3. The ADAT3 from *Mus musculus* (MmADAT3,

NP_001094076.1), *Danio rerio* (DrADAT3, NP_001005300.2), *Branchiostoma japonicum* (BjADAT3), *Mizuhopecten yessoensis* (MyADAT3, XP_021339414.1) and *Trypanosoma brucei* (TbADAT3, XP_829586.1) were included. The secondary structure elements were labeled above the sequences (based on MmADAT3). The red dots indicated the residues of the pseudo-catalytic site of ADAT3. The positively charged (rich of K and R) region was indicated by the green box.

Supplementary Figure 3

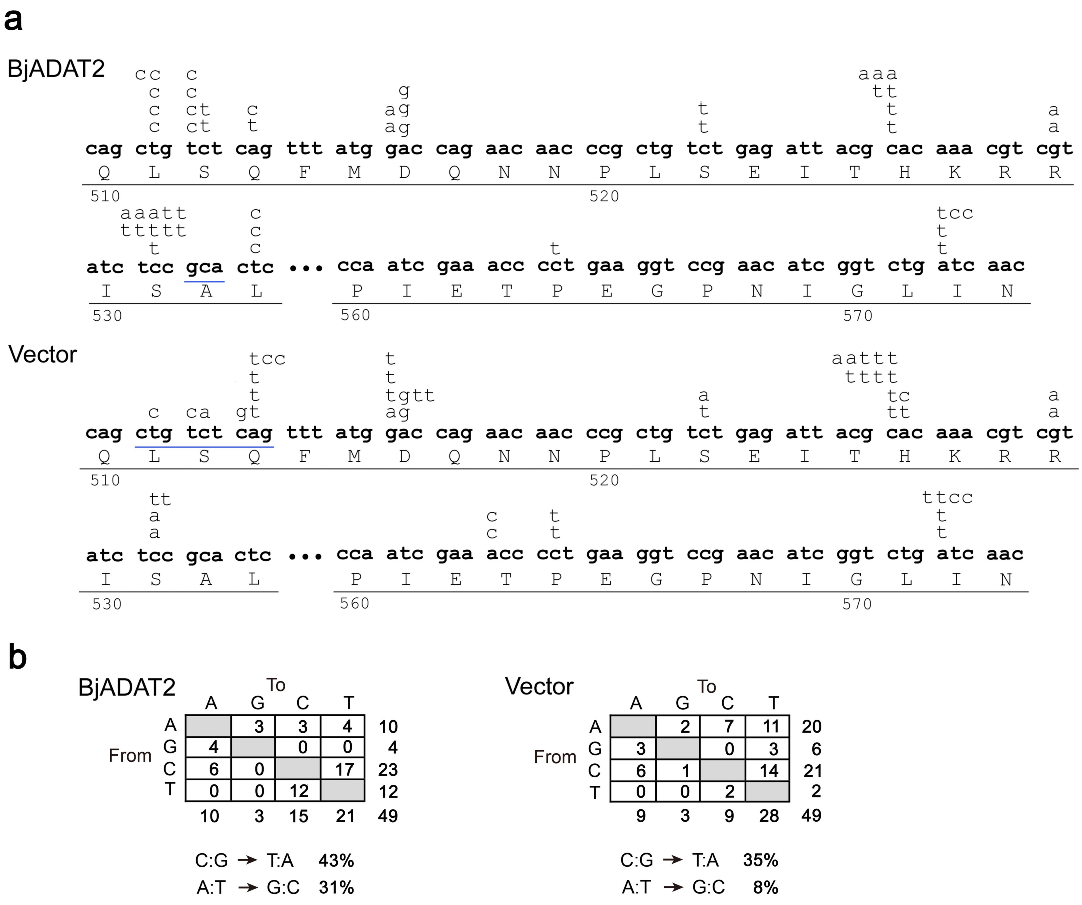

BjADAT2 induced mutagenesis in *E. coli alkA*⁻ strain. (**a**) Comparison of the distribution of independent *rpoB* mutations identified in Rif^{R} colonies obtained from BjADAT2- and empty vector-transformants (50 independent cultures for each group, and one clone per culture). The sequence shown here contains all of the mutations detected in this assay. Deletion sites were underlined in blue. Each clone contains one and only one of the mutations. (**b**) Comparison of the pattern of base substitutions.

Supplementary Figure 4

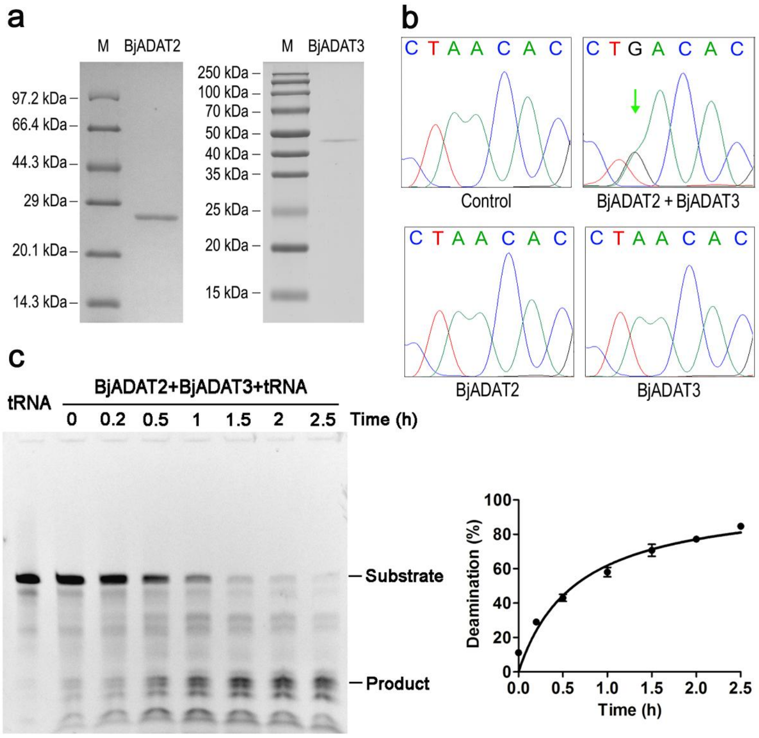

tRNA editing activity of BjADAT2 and BjADAT3. (**a**) SDS-PAGE of recombinant proteins BjADAT2 and BjADAT3. The purified His-tagged BjADAT2 and BjADAT3 both yielded a single band of approximately 26.1 and 46.7 kDa, respectively, well matching the expected sizes. (**b**) Chromatogram obtained after sequencing of the anticodon loop of *in vitro* transcribed $tRNA^{Val}_{(AAC)}$ incubated with purified BjADAT2, BjADAT3, BjADAT2 plus BjADAT3, or without these proteins (control). After incubated with BjADAT2 plus BjADAT3, the 'A' at the first position of the anticodon was largely replaced by a 'G' (indicated by a green arrow). (**c**) Representative TBE-Urea gel and kinetic analysis of $tRNA^{Val}_{(AAC)}$ deamination by BjADAT2/3 complex. The fraction of deaminated tRNA were plotted as a function of time and fit to a single exponential equation to extract k_{app}. Data are represented as the mean ± SEM from three independent experiments. The gels shown in **a**, **c** are raw images.

Supplementary Figure 5

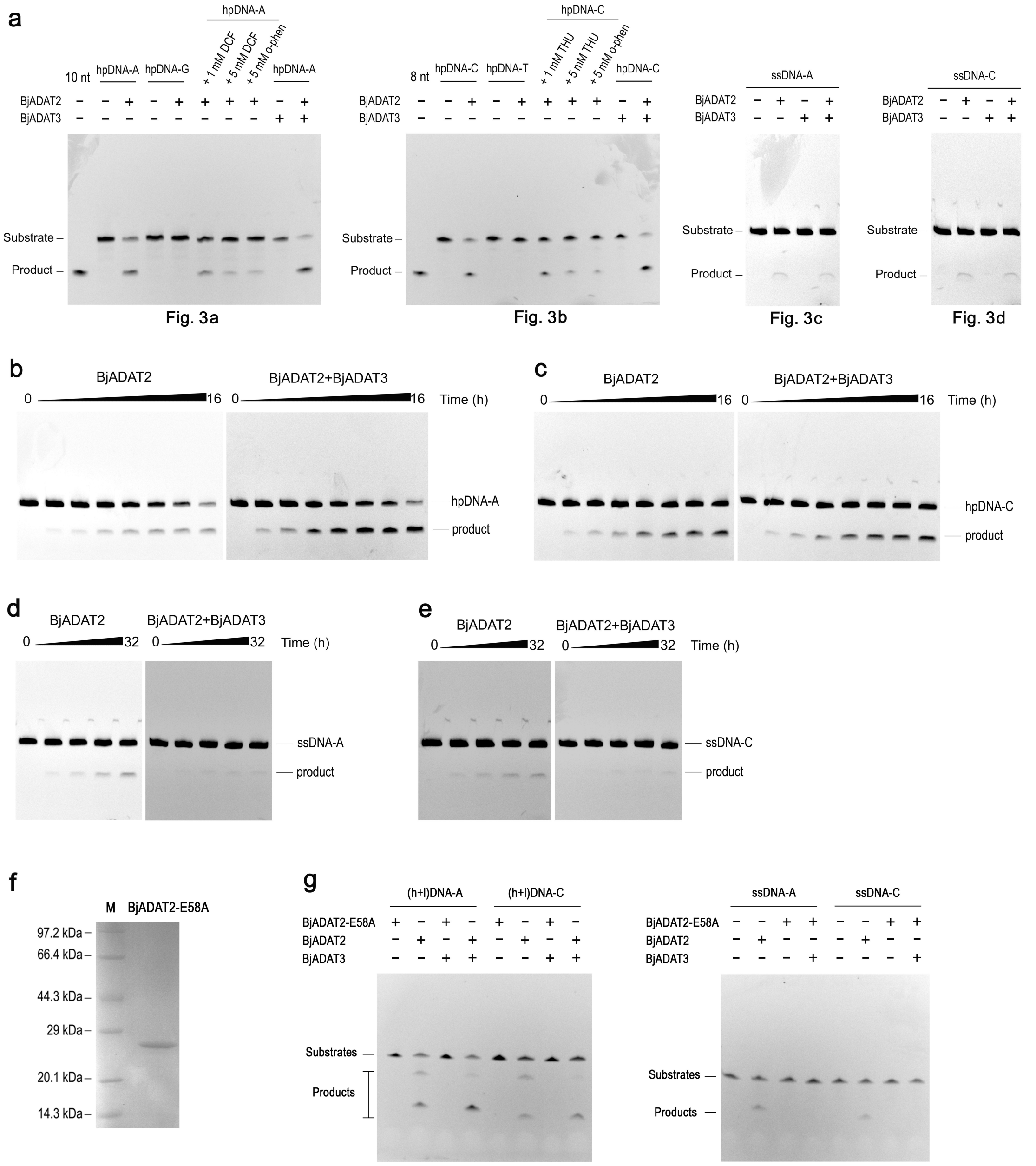

Original source images for data in Figure 3. (**a**) Raw gel images of Fig. 3a-3d. (**b-e**) Representative TBE-Urea gels showing the time course of substrate deamination for the data in Fig. 3e. The two hairpin-structure substrates hpDNA-A (**b**) and hpDNA-C (**c**), as well as the two linear substrates ssDNA-A (**d**) and ssDNA-C (**e**), were incubated with BjADAT2 in the presence or absence of BjADAT3 at 28 °C for 0-16 h or 0-32 h, respectively. (**f**) SDS-PAGE of the recombinant protein BjADAT2-E58A. The purified His-tagged BjADAT2-E58A yielded a single band of approximately 26 kDa, well matching the expected sizes. (**g**) TBE-Urea gel of the deamination assay showing that no product band was seen in the lane of substrates treated with BjADAT2-E58A for 20 h. The gels shown in **b-g** are raw images.

Supplementary Figure 6

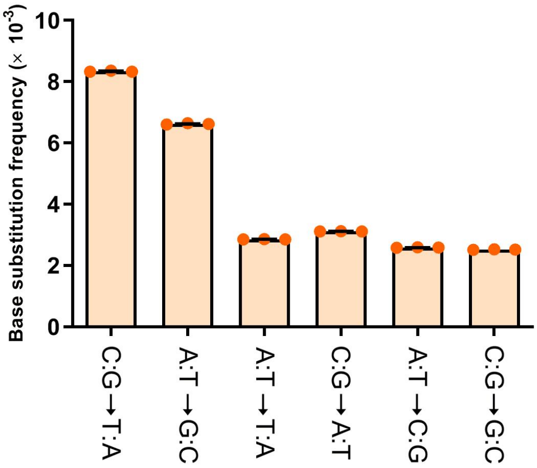

Somatic base-substitution mutation frequency in amphioxus. The somatic mutations calling from the whole-genome re-sequencing data of *Branchiostoma belcheri* muscular tissues (NCBI SRA accession: SRR1174914, SRR1174915 and SRR1174916, from three individuals, respectively[1]) was preformed according to the method of Bi et al., 2020[1]. The base-substitution frequency of each type was calculated by the ratio of the number of observed base substitutions to the number of corresponding nucleotide sites analyzed. For example, C:G to T:A mutation frequency was calculated by the ratio of the total number of C to T plus G to A mutations to the total number of C plus G sites in the reference genome of *B. belcheri* v. 18h27 (GenBank assembly accession: GCF_001625305.1). Data are represented as the mean ± SEM from three independent individuals of amphioxus.

Supplementary Figure 7

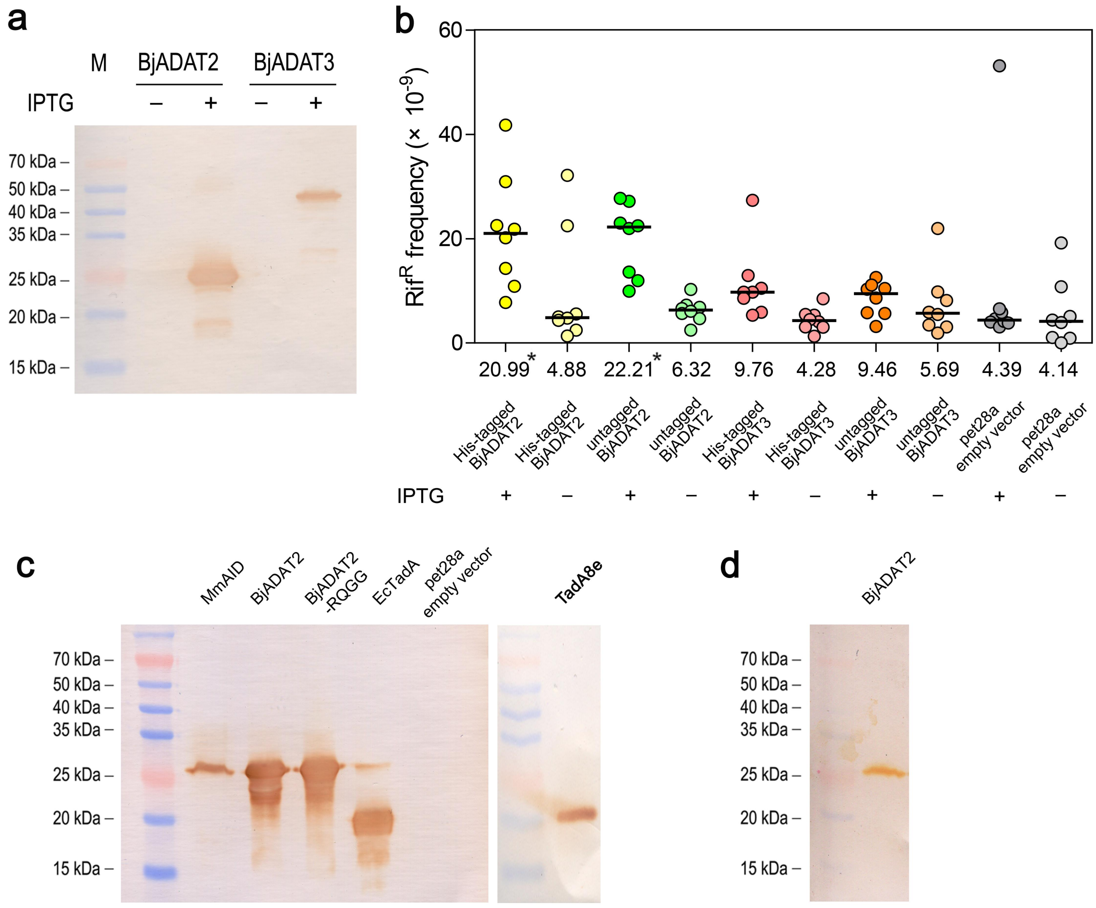

Expression levels and mutagenic activities of BjADAT2 and BjADAT3 in *E. coli.* (**a**) Western blot analysis of the expression of the His-tagged BjADAT2 and BjADAT3 in *E. coli* induced with IPTG (+) or not (-) for 24 h. The bacteria samples were boiled in SDS-PAGE loading buffer and run on a 12% SDS-PAGE gel. The gel was transferred to a PVDF membrane by semi-dry transfer. The primary antibody was mouse anti-His-tag monoclonal antibody (1:5000; CWBIO, China), and the secondary antibody was goat anti-mouse IgG antibody-HRP (1:8000; CWBIO). The bands were visualized using diaminobenzidine (DAB) kit (CWBIO). The predicted sizes for N-terminal His-tagged BjADAT2 and BjADAT3 are 26.1 and 46.7 kDa, respectively. They both matched the expected sizes, indicating they were correctly expressed. (**b**) Rif^R frequencies of the *E. coli* transformed with pet28a-BjADAT2, pet28a-BjADAT3 or pet28a empty vector in the presence of IPTG (+) or not (-). The horizontal bars represent the median (numerical value below each column), and statistical differences of each deaminase compared to empty pet28a vector were assessed using a two-tailed Willcoxon rank sum for unpaired data, $*p < 0.05$. In the presence of the transcriptional inducer IPTG, the BjADAT2-transformed *E. coli* generated Rif^R colonies at a median frequency that was ~5 times higher than that of vector-transformed controls. The untagged BjADAT2 increased mutation to Rif^R to levels comparable with those achieved by N-terminal His-tagged BjADAT2, indicating that the N-terminal His-tag had no effects on the mutagenic activity of BjADAT2. In addition, neither His-tagged nor untagged BjADAT3 exhibited a notably mutagenic activity. (**c**) Western blot analysis of the expression of the

His-tagged deaminases in *E. coli* induced with IPTG for 24 h. The western blots were performed as described above. The predicted sizes for His-tagged MmAID, BjADAT2, BjADAT2-RQGG, EcTadA and TadA8e are 27, 26, 26, 22 and 22 kDa, respectively. All of the His-tagged proteins matched the expected sizes. (**d**) Western blot analysis of the expression of the His-tagged BjADAT2 in *E. coli alkA*$^{-}$ cells induced with IPTG for 24 h. The protein matched the expected size.

Supplementary Figure 8

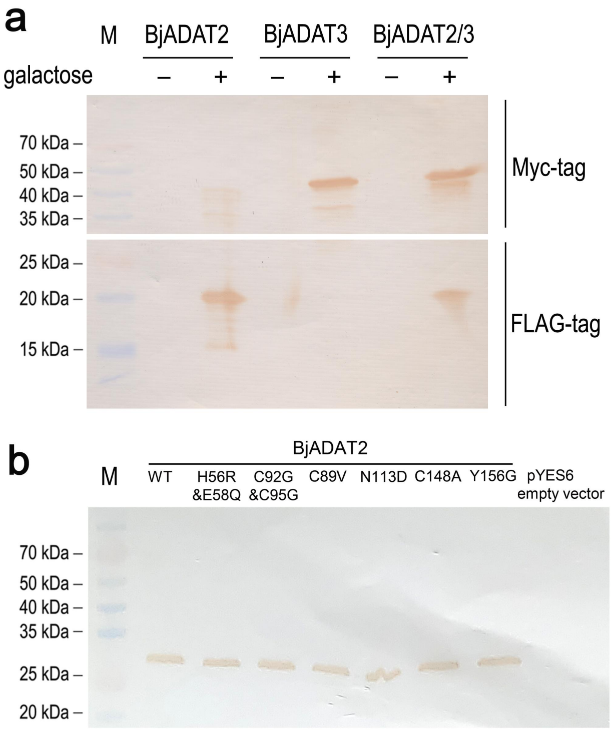

Expression levels of BjADAT2 and BjADAT3 in *S. cerevisiae.* (**a**) Western blot analysis of the expression of the C-terminal Flag-tagged BjADAT2 or C-terminal Myc-tagged BjADAT3 in BY4741 induced with galactose (+) or not (-) for 3 days. The yeast cells were disrupted by vortexing with the glass beads. Cell debris was removed by centrifugation, and the supernatant was run on a 12% SDS-PAGE gel. Western blots were performed as described above, except that the primary antibody was mouse anti-Flag-tag monoclonal antibody (1:3000; Beyotime, China) or mouse anti-Myc-tag monoclonal antibody (1:3000; Beyotime) as indicated. Both of the Flag-tagged BjADAT2 and Myc-tagged BjADAT3 proteins matched the expected sizes (20.9 and 44.5 kDa, respectively), indicating they were correctly expressed. (**b**) Western blot analysis of the expression of BjADAT2 mutants (carrying a C-terminal His-tag) in yeast cells induced with galactose for 3 days. The primary antibody was mouse anti-His-tag monoclonal antibody (1:5000; CWBIO, China), and the secondary antibody was goat anti-mouse IgG antibody-HRP (1:8000; CWBIO).

Supplementary Figure 9

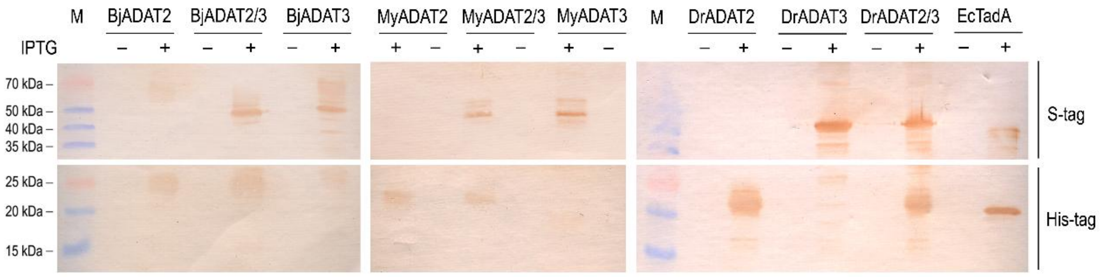

Expression levels of recombinant deaminase proteins in *E. coli.* Western blot analysis of the expression of the His-tagged or S-tagged deaminases in *E. coli* induced with IPTG (+) or not (-) for 24 h. The western blots were performed as described above, except that the primary antibody was mouse anti-His-tag monoclonal antibody (1:5000; CWBIO) or mouse anti-S-tag monoclonal antibody (1:5000; Sangon Biotech, China) as indicated. The predicted sizes for His-tagged BjADAT2, MyADAT2, DrADAT2, and EcTadA are 24.1, 20.8, 24.8, and 20.5 kDa, respectively. The predicted sizes for S-tagged BjADAT3, MyADAT3, and DrADAT3 are 47.0, 48.7, and 41.8 kDa, respectively. All of the His- or S-tagged proteins matched the expected sizes, indicating they were correctly expressed.

Supplementary Table 1. Sequence of *can1* gene mutation in yeast BY4741 strain

Position	Mutation	Flanking Sequence [a]	Hairpin
Expression of BjADAT2			
296	G>A	TGATTGCCCTTG **G** TGGTACTATTGG	-
301	A>C	GCCCTTGGTGGT **A** CTATTGGTACAG	-
353	G>A	TGACCAACGCCG **G** CCCAGTGGGCGC (2)	+
449	Deletion	TCATCCCTGTTA **~~C~~** ATCCTCTTTCAC	-
509	G>A	GTGCGGCCAATG **G** TTACATGTATTG	-
679	G>T	TACGGTGAATTC **G** AGTTCTGGGTCG	-
732	C>G	GTTTCTAATATA **C** TGTTTTTGTATG	-
763	A>G	GGTGCTGGGGTT **A** CCGGCCCAGTTG	+
939	Deletion	AGCTGCAAACCC **~~C~~** AGAAAATCCGTT	-
1098	T>G	TATTATTGCTAT **T** GAGAACTCTGGT	-
1196	G>A	ACGTTGGTTCCC **G** TATTTTATTTGG	-
1227	Insertion	AGAACAAGTT **(39bp)** GGCTCCTAAA	-
1244	C>G	CTAAATTCCTGT **C** AAGGACCACCAA	-
1256	A>G	CAAGGACCACCA **A** AGGTGGTGTTCC	+
1335	Deletion	ATCTACTGGTGG **~~T~~** GACAAAGTTTTC	-
1728	Insertion	ATGGGAAGATCA **(T)** TGAACCAAAGAC	-
Co-expression of BjADAT2 and BjADAT3			
353	G>A	TGACCAACGCCG **G** CCCAGTGGGCGC	+
537	C>T	TTCTTGGGCAAT **C** ACTTTTGCCCTG	+
625	Insertion	TTAGTATTTTTT **(T)** GGGTAATTATCA	-
763	A>G	GGTGCTGGGGTT **A** CCGGCCCAGTTG	+
801	T>A	GAGAAACCCAGG **T** GCCTGGGGTCCA	+
809	G>C	CAGGTGCCTGGG **G** TCCAGGTATAAT	+
1072	T>A	ACTTCCTACGTT **T** CTACTTCTCCCT	-
1286	Deletion	GCAGTTTTCG **~~(TTACTGCT…CG, 63bp)~~** AAT	+
1361	Deletion	AATGGCTATTAA **~~A~~** TATCACTGGTGT	-
1456	Deletion	GGCATCTCTCGT **~~G~~** ACGAGTTACCAT	-
1622	G>A	TCTTAGCTGTTT **G** GATCTTATTTCA	-
Empty vector control			
343	A>C	TCCACACCTCTG **A** CCAACGCCGGCC	-
374	C>A	GCGCTCTTATAT **C** ATATTTATTTAT	-
610	T>C	CCACTGGCGGCA **T** GGATTAGTATTT	-
627	G>A	TAGTATTTTTTG **G** GTAATTATCACA	-
718	G>A	TTAGCCATTATC **G** GGTTTCTAATAT	-
938	C>T	AAGCTGCAAACC **C** CAGAAAATCCGT	-
1262	G>A	CCACCAAAGGTG **G** TGTTCCATACAT	-

1391	G>A	GCTTTTTTGCAT **G** GTTATTTATCTC	-
1612	Deletion	ATTTTCCTGTTC **~~T~~** TAGCTGTTTGGA	-
1616	C>G	TCCTGTTCTTAG **C** TGTTTGGATCTT	-
1622	G>A	TCTTAGCTGTTT **G** GATCTTATTTCA	-

[a] Mutated nucleotides are marked in green. The hairpin loops and stems are marked in blue and red, respectively.

Supplementary Table 2. Sequences of oligonucleotides used in this study

Description	Sequence (5' to 3')	Remarks
Primer pair for cloning partial cDNA fragments of BjADAT2	S: ATGGCGGACCAGGGTGAGAT<br>AS: CTAGAACTCCGTGTAGCCATACCCA	
Primer for 5' RACE of BjADAT2	CCCTGTGCTACTATCTGTCCTTG	SMARTer RACE 5'/3' Kit (Clontech)
Primer for 3' RACE of BjADAT2	GGGATGGTTCAGAGTACTATGC	SMARTer RACE 5'/3' Kit (Clontech)
Primer pair for cloning the ORF of BjADAT2	S: ATGGCGGACCAGGGTGAGATC<br>AS: CTAGAACTCCGTGTAGCCATAC	
Primer pair for cloning partial cDNA fragments of BjADAT3	S: CCCCACCCTGTACCTGTCCT<br>AS: TGGTTGATGTTAGGCTGGGTG	
Primer for 5' RACE of BjADAT3	AGACGGGATGTTTGCTTCTTGTC	SMARTer RACE 5'/3' Kit (Clontech)
Primer for 3' RACE of BjADAT3	GTGTACTGGATATGACTTGTATGTG	SMARTer RACE 5'/3' Kit (Clontech)
Primer pair for cloning the ORF of BjADAT3	S: ATGGCGGCGCCCATGAGTGATTT<br>AS: TTAAACAGTCTCTAAGTC	
Primer pair for subcloning BjADAT2 into pet28a vector (carrying a N-terminal His-tag)	S: GGAATTCATGGCGGACCAGGGTGAGATCGAG<br>AS: CCGCTCGAGTTCAGAACTCCGTGTAGCCATA C	The EcoR I and Xho I restriction sites were underlined.
Primer pair for subcloning BjADAT2 into pet28a vector (lacking the N-terminal His-tag)	S: CATGCCATGGCGGACCAGGGTGAGATCGAG<br>AS: CCGCTCGAGTTCAGAACTCCGTGTAGCCATA C	The Nco I and Xho I restriction sites were underlined.
Primer pair for subcloning BjADAT3 into pet28a vector (carrying a N-terminal His-tag)	S: GGAATTCATGGCGGCGCCCATGAGTGATTT<br>AS: CCGCTCGAGTTTAAACAGTCTCTAAGTC	The EcoR I and Xho I restriction sites were underlined.
Primer pair for subcloning BjADAT3 into pet28a vector (lacking the N-terminal His-tag)	S: CATGCCATGGCGGCGCCCATGAGTGATTT<br>AS: CCGCTCGAGTTTAAACAGTCTCTAAGTC	The Nco I and Xho I restriction sites were underlined.
Two pairs of primer for constructing BjADAT2-RQGG mutant on the basis of pet28a-BjADAT2	S1: GTCCGTGCGCAGATGGTAGCGGTTGGCCAGC TGCTGG<br>AS1: CTACCATCTGCGCACGGACTGTAGCATTTTT TGACTCG<br>S2: CCGGTATCATGGGTGCCGGCGCGCTCAGACTG CTCAAC<br>AS2: CGGCACCCATGATACCGGGCTCACACGTCA CGTAC	Mut Express II Fast Mutagenesis Kit (Vazyme, China)
Primer pair for constructing BjADAT2-E58A mutant on the basis of pet28a-BjADAT2	S: CAAAAAATGCTACAGTCCATGCGGCGATGGTA GCGGTTG<br>AS: ATGGACTGTAGCATTTTTTGACTCGTTAACTC TGTTGTG	Mut Express II Fast Mutagenesis Kit (Vazyme, China)
Primer pair for cloning MmAID	S: ATGGACAGCCTTCTGATGAAGC	

	AS: TCAAAATCCCAACATACGAAATGC	
Primer pair for subcloning MmAID into pet28a vector	S: GGAATTCATGGACAGCCTTCTGATG<br>AS: CCGCTCGAGTTCAAAATCCCAACATACGA	The EcoR I and Xho I restriction sites were underlined.
Primer pair for cloning EcTadA	S: ATGTCCGAAGTCGAGTTTTCC<br>AS: GTCAGTAGAGGATTGTGCTTTTTTC	
Primer pair for subcloning EcTadA into pet28a vector	S: GGAATTCATGTCCGAAGTCGAGTTTTCC<br>AS: CCCAAGCTTGCTAGTCAGTAGAGGATTGTG	The EcoR I and Hind III restriction sites were underlined.
Primer pair for cloning TadA7.10	S: GGTGGTTCTTCCGAAGTCGAG<br>AS: GCCGATGGCTAAACCAATAGAATAC	
Primer pairs for site-directed mutation (from TadA7.10 to TadA8e)	S1: GGTGCCGCAGGTTCACTGATGAACGTGCTGAATTACCCAGGCA<br>AS1: CATCAGTGAACCTGCGGCACCACGCTTGGAGTTGCGAACACC<br>S2: GAATGTGCGGCGCTGTTGTGTGACTTTTATCGCATGCCCAGG<br>AS2: ACACAACAGCGCCGCACATTCGTCCGCCAATATGCCTTCTG	Mut Express II Fast Mutagenesis Kit (Vazyme, China)
Primer pair for subcloning TadA8e into pet28a vector	S: GGAATTCATGTCCGAAGTCGAGTTTTCC<br>AS: CCCAAGCTTGCTAGTTAATAGAGGATTGTGCTT	The EcoR I and Hind III restriction sites were underlined.
Synthetic ssDNA according to amphioxus $tRNA^{Val}_{(AAC)}$	CCGTGGTGTAGCGGTTATCACATCTGCCTAACACGCAGAAGGTCCCCGGTTCGATCCCGGGCGGAATCA	
tRNA-S	AATTGGGCCCGATTCCGTGGTGTAGCGGTTA	The Apa I restriction site was underlined.
tRNA-AS	GGAATTCCATATGTGATTCCGCCCGGGAT	The Nde I restriction site was underlined.
Primer pair for subcloning BjADAT2 into pcDNA3.1-GFP vector	S: CCCAAGCTTGCCACCATGGCGGACCAGGGTGAGATC<br>AS: CCGCTCGAGGAACTCCGTGTAGCCATACC	The Hind III and Xho I restriction sites were underlined.
Primer pair for subcloning BjADAT3 into pcDNA3.1-mCherry vector	S: CCCAAGCTTGCCACCATGGCGGCGCCCATGAGTGATTT<br>AS: CCGCTCGAGAACAGTCTCTAAGTCTAAAGAG	The Hind III and Xho I restriction sites were underlined.
Primer pair for subcloning BjADAT2 into pESC vector (carrying a C-terminal Flag-tag)	S: GGAATTCATGGCGGACCAGGGTGAGATCGAG<br>AS: CGGACTAGTGCGTTCCCTCCCAGTCCTGTACTT	The EcoR I and Spe I restriction sites were underlined.
Primer pair for subcloning BjADAT2 into pESC vector (insertion into the EcoR I site; lacking the C-terminal Flag-tag)	S: AATTTTTGAAAATTCGAATTCATGGCGGACCAGGGTGAG<br>AS: CCTTTAGTGAGGGTTGAATTCGAACTCCGTGTAGCCATACCCA	ClonExpress II One Step Cloning Kit (Vazyme, China)
Primer pair for subcloning BjADAT3 into pESC vector (insertion into the Sal I site;	S: CTATAGGGCCCGGGCGTCGACATGGCGGCGCCCATGAGT<br>AS: CAACTTCTGTTCCATGTCGACAACAGTCTCTA	ClonExpress II One Step Cloning Kit (Vazyme, China)

carrying a C-terminal myc-tag)	AGTCTAAAGAGCTGCA	
Primer pair for subcloning BjADAT3 into pESC vector (insertion into the Sal I site; lacking the C-terminal myc-tag)	S: CTATAGGGCCCGGGCGTCGACATGGCGGCGCCCATGAGT<br>AS: CAACTTCTGTTCCATGTCGACTTAAACAGTCTCTAAGTCTAAAGAGCTGCA	ClonExpress II One Step Cloning Kit (Vazyme, China)
Primer pair for subcloning BjADAT2 into pYES6 vector (not carrying any tags)	S: CCCAAGCTTGCCACCATGGCGGACCAGGGTGAGATC<br>AS: CCGCTCGAGTTCAGAACTCCGTGTAGCCATAC	The Hind III and Xho I restriction sites were underlined.
Primer pair for constructing C89V mutant on the basis of pYES6-BjADAT2	S: GTTGAGCCCTGTATCATGTGTGCC<br>AS: CGTCACGTACAGCTGGACGATC	MutanBEST Kit (TaKaRa)
Primer pair for constructing C148A mutant on the basis of pYES6-BjADAT2	S: GCTGCAGCCTTACTGAAACAGTTC<br>AS: CGGCTCTGCAAAGATGCCACCAA	MutanBEST Kit (TaKaRa)
Primer pair for constructing N113D mutant on the basis of pYES6-BjADAT2	S: GACCCCCGCTTCGGGGGCTGT<br>AS: GGGGCATCCGTACCACACCAC	MutanBEST Kit (TaKaRa)
Primer pair for constructing Y156G mutant on the basis of pYES6-BjADAT2	S: GGTGGGCAGCAGAACCCTAATGC<br>AS: GAACTGTTTCAGTAAGGCTGCAC	MutanBEST Kit (TaKaRa)
Primer pair for constructing H56R&E58Q mutant on the basis of pYES6-BjADAT2	S: GTCCGTGCGCAGATGGTAGCGGTTGGCCAGCTGCTGG<br>AS: CTACCATCTGCGCACGGACTGTAGCATTTTTTGACTCG	Mut Express II Fast Mutagenesis Kit (Vazyme, China)
Primer pair for constructing C92G&C95G mutant on the basis of pYES6-BjADAT2	S: CCGGTATCATGGGTGCCGGCGCGCTCAGACTGCTCAAC<br>AS: CGGCACCCATGATACCGGGCTCACACGTCACGTAC	Mut Express II Fast Mutagenesis Kit (Vazyme, China)
Primer pair for deleting the termination codon of BjADAT2 and its mutants in the pYES6 vector (carrying a C-terminal His-tag)	S: ACGGAGTTCTGGCTCGAGTCTAGAGGGCCCTTC<br>AS: CTCGAGCCAGAACTCCGTGTAGCCATACCCAGC	Mut Express II Fast Mutagenesis Kit (Vazyme, China)
Primer pair for subcloning BjADAT2 into petDuet vector (insertion into the EcoR I site; carrying a N-terminal His-tag)	S: CCACAGCCAGGATCCGAATTCGATGGCGGACCAGGGTGAG<br>AS: AGGCGCGCCGAGCTCGAATTCGAACTCCGTGTAGCCATACCCA	ClonExpress II One Step Cloning Kit (Vazyme, China)
Primer pair for subcloning BjADAT3 into petDuet vector (insertion into the Xho I site; carrying a C-terminal S-tag)	S: GCTGACGTCGGTACCCTCGAGATGGCGGCGCCCATGAGT<br>AS: GGTTTCTTTACCAGACTCGAGAACAGTCTCTAAGTCTAAAGAGCTGCA	ClonExpress II One Step Cloning Kit (Vazyme, China)
Primer pair for cloning DrADAT2	S: ATGCAAGAAGTCGGTGTTGAT<br>AS: TCAGCACACAGCAGATGGGGA	

Primer pair for subcloning DrADAT2 into petDuet vector (insertion into the EcoR I site; carrying a N-terminal His-tag)	S: CCACAGCCAGGATCCGAATTCGATGCAAGAAGTCGGTGTTGATC<br>AS: AGGCGCGCCGAGCTCGAATTCTCAGCACACAGCAGATGGGG	ClonExpress II One Step Cloning Kit (Vazyme, China)
Primer pair for cloning DrADAT3	S: ATGGAGCCCCAAGCCAAAC<br>AS: TTACTCTTTACACAAATCCTCACAC	
Primer pair for subcloning DrADAT3 into petDuet vector (insertion into the Xho I site; carrying a C-terminal S-tag)	S: GCTGACGTCGGTACCCTCGAGATGGAGCCCCAAGCCAAA<br>AS: GGTTTCTTTACCAGACTCGAGCTCTTTACACAAATCCTCACACGC	ClonExpress II One Step Cloning Kit (Vazyme, China)
Primer pair for cloning MyADAT2	S: ATGGATGAAATTCATCAGAAGTGG<br>AS: TTAATCTGTGTTTTTAATCTTTCGC	
Primer pair for subcloning MyADAT2 into petDuet vector (insertion into the EcoR I site; carrying a N-terminal His-tag)	S: CCACAGCCAGGATCCGAATTCGATGGATGAAATTCATCAGAAGTGG<br>AS: AGGCGCGCCGAGCTCGAATTCTTAATCTGTGTTTTTAATCTTTCGCTT	ClonExpress II One Step Cloning Kit (Vazyme, China)
Primer pair for cloning MyADAT3	S: ATGAACACCAGACACCCTGTGG<br>AS: TTATGTTACAGCAGCATCATACAGTTG	
Primer pair for subcloning MyADAT3 into petDuet vector (insertion into the Xho I site; carrying a C-terminal S-tag)	S: GCTGACGTCGGTACCCTCGAGATGAACACCAGACACCCTGTGG<br>AS: GGTTTCTTTACCAGACTCGAGTGTTACAGCAGCATCATACAGTTGA	ClonExpress II One Step Cloning Kit (Vazyme, China)
Primer pair for subcloning EcTadA into petDuet vector (insertion into the EcoR I site; carrying a N-terminal His-tag)	S: CCACAGCCAGGATCCGAATTCGATGTCCGAAGTCGAGTTTTCC<br>AS: AGGCGCGCCGAGCTCGAATTCCTAGTCAGTAGAGGATTGTG	ClonExpress II One Step Cloning Kit (Vazyme, China)
rpoB-S	TTGGCGAAATGGCGGAAAACC	
rpoB-AS	CACCGACGGATACCACCTGCTG	
gyrA-S	GCGCGGCTGTGTTATAATTT	
gyrA-AS	TTCCGTGCCGTCATAGTTATC	
CAN-S	CAGACTTCTTAACTCCTG	
CAN-AS	GAAATGTGATCAAAGGTAATAAAACG	
CAN-P1	GGAACTTTGTACGTCCAAAATTG	
CAN-P2	GGAACTTAGTGTAGTTGG	

The ‘S’ means sense primer, and the ‘AS’ means antisense primer.

Supplementary References

1. Bi C, Lu N, Huang Z, Chen J, He C, Lu Z. Whole-genome resequencing reveals the pleistocene temporal dynamics of *Branchiostoma belcheri* and *Branchiostoma floridae* populations. *Ecology and Evolution* **10**, 8210-8224 (2020).
