# Supplementary material for: Amphioxus adenosine-to-inosine tRNA-editing enzyme that can perform C-to-U and A-to-I deamination of DNA
Source: Commun Biol. 2023 Jul 18;6:744. doi: 10.1038/s42003-023-05134-0 (PMC10354150; doi:10.1038/s42003-023-05134-0)
Supplement: Supplementary file 5 — Reporting Summary [file 42003_2023_5134_MOESM5_ESM.pdf]

nature portfolio

Corresponding author(s): Zhan Gao

Last updated by author(s): Jun 28, 2023

Reporting Summary

Nature Portfolio wishes to improve the reproducibility of the work that we publish. This form provides structure for consistency and transparency in reporting. For further information on Nature Portfolio policies, see our Editorial Policies and the Editorial Policy Checklist.

Statistics

For all statistical analyses, confirm that the following items are present in the figure legend, table legend, main text, or Methods section.

n/a	Confirmed	
☐	☒	The exact sample size (*n*) for each experimental group/condition, given as a discrete number and unit of measurement
☐	☒	A statement on whether measurements were taken from distinct samples or whether the same sample was measured repeatedly
☐	☒	The statistical test(s) used AND whether they are one- or two-sided<br>*Only common tests should be described solely by name; describe more complex techniques in the Methods section.*
☒	☐	A description of all covariates tested
☒	☐	A description of any assumptions or corrections, such as tests of normality and adjustment for multiple comparisons
☐	☒	A full description of the statistical parameters including central tendency (e.g. means) or other basic estimates (e.g. regression coefficient) AND variation (e.g. standard deviation) or associated estimates of uncertainty (e.g. confidence intervals)
☐	☒	For null hypothesis testing, the test statistic (e.g. *F*, *t*, *r*) with confidence intervals, effect sizes, degrees of freedom and *P* value noted<br>*Give P values as exact values whenever suitable.*
☒	☐	For Bayesian analysis, information on the choice of priors and Markov chain Monte Carlo settings
☒	☐	For hierarchical and complex designs, identification of the appropriate level for tests and full reporting of outcomes
☒	☐	Estimates of effect sizes (e.g. Cohen's *d*, Pearson's *r*), indicating how they were calculated

Our web collection on statistics for biologists contains articles on many of the points above.

Software and code

Policy information about availability of computer code

Data collection	Homology searches in the GenBank database were carried out by BLASTP network server (https://blast.ncbi.nlm.nih.gov/Blast.cgi) at the NCBI. The three-dimensional (3D) structure of BjADAT2 was predicted using the iterative threading assembly refinement (I-TASSER) program (https://zhanglab.ccmb.med.umich.edu/I-TASSER/). Hairpin structure around the mutation site was predicted by UNAFold Web Server (http://www.unafold.org/).
Data analysis	Multiple alignments of the protein sequences was generated using ESPript 3.x software (http://espript.ibcp.fr/ESPript/cgi-bin/ESPript.cgi).<br>In the E. coli Cirseq and rare-mutation analysis, fastp v1.0 was used to trim library adaptors and to remove reads with quality scores < 20. The python scripts modified from Acevedo et al. (Nature, 2014, 505:686-690) were used to analyze the clean data, and all statistics were done in R 3.6.3.

For manuscripts utilizing custom algorithms or software that are central to the research but not yet described in published literature, software must be made available to editors and reviewers. We strongly encourage code deposition in a community repository (e.g. GitHub). See the Nature Portfolio guidelines for submitting code & software for further information.

Data

Policy information about availability of data

All manuscripts must include a data availability statement. This statement should provide the following information, where applicable:

- Accession codes, unique identifiers, or web links for publicly available datasets
- A description of any restrictions on data availability
- For clinical datasets or third party data, please ensure that the statement adheres to our policy

Raw reads of Cirseq were deposited in NCBI Sequence Read Archive with BioProject no. PRJNA753282.

Research involving human participants, their data, or biological material

Policy information about studies with human participants or human data. See also policy information about sex, gender (identity/presentation), and sexual orientation and race, ethnicity and racism.

Reporting on sex and gender	n/a
Reporting on race, ethnicity, or other socially relevant groupings	n/a
Population characteristics	n/a
Recruitment	n/a
Ethics oversight	n/a

Note that full information on the approval of the study protocol must also be provided in the manuscript.

Field-specific reporting

Please select the one below that is the best fit for your research. If you are not sure, read the appropriate sections before making your selection.

☒ Life sciences ☐ Behavioural & social sciences ☐ Ecological, evolutionary & environmental sciences

For a reference copy of the document with all sections, see nature.com/documents/nr-reporting-summary-flat.pdf

Life sciences study design

All studies must disclose on these points even when the disclosure is negative.

Sample size	n/a
Data exclusions	No Data was excluded
Replication	Representative experiments have been repeated at least two to three times and all replicates were successful.
Randomization	n/a
Blinding	n/a

Reporting for specific materials, systems and methods

We require information from authors about some types of materials, experimental systems and methods used in many studies. Here, indicate whether each material, system or method listed is relevant to your study. If you are not sure if a list item applies to your research, read the appropriate section before selecting a response.

Materials & experimental systems

n/a	Involved in the study	
☐	☒	Antibodies
☐	☒	Eukaryotic cell lines
☒	☐	Palaeontology and archaeology
☐	☒	Animals and other organisms
☒	☐	Clinical data
☒	☐	Dual use research of concern
☒	☐	Plants

Methods

n/a	Involved in the study	
☒	☐	ChIP-seq
☒	☐	Flow cytometry
☒	☐	MRI-based neuroimaging

Antibodies

Antibodies used	mouse anti-Flag-tag monoclonal antibody (Beyotime, China, AF519)<br>mouse anti-Myc-tag monoclonal antibody (Beyotime, China, AM926)<br>mouse anti-S-tag monoclonal antibody (Sangon Biotech, China, D191105)<br>mouse anti-His-tag monoclonal antibody (CWBIO, China, CW0286)<br>HRP-conjugated goat anti-mouse IgG antibody (CWBIO, China, CW0102)
Validation	Validation by manufacturer as detailed on their website.

Eukaryotic cell lines

Policy information about cell lines and Sex and Gender in Research

Cell line source(s)	HEK293T cell line was a gift from Dr. Jianfeng Zhou, Ocean University of China.
Authentication	The cell lines were authenticated.
Mycoplasma contamination	All cell lines tested negative for mycoplasma contamination.
Commonly misidentified lines<br>(See ICLAC register)	No misidentified lines were used in this study.

Animals and other research organisms

Policy information about studies involving animals; ARRIVE guidelines recommended for reporting animal research, and Sex and Gender in Research

Laboratory animals	Amphioxus (Branchiostoma japonicum), adult;<br>Zebrafish (Danio rerio), 3 months;<br>Japanese scallops (Mizuhopecten yessoensis), adult;<br>Mouse (Mus musculus), 11 months.
Wild animals	The study did not involve wild animals.
Reporting on sex	n/a; In this study, all of the animals were only used for cloning deaminase genes.
Field-collected samples	This study did not involve sample collected from the field.
Ethics oversight	All animals were maintained followed the ethical guidelines, and all experimental protocols were approved by the Institutional Animal Care and Use Committee of the Ocean University of China.

Note that full information on the approval of the study protocol must also be provided in the manuscript.
